# A Deep Learning Approach for Accurate Path Loss Prediction in LoRaWAN Livestock Monitoring

**DOI:** 10.3390/s24102991

**Published:** 2024-05-08

**Authors:** Mike O. Ojo, Irene Viola, Silvia Miretti, Eugenio Martignani, Stefano Giordano, Mario Baratta

**Affiliations:** 1 Department of Biological and Agricultural Engineering, Texas A&M AgriLife Research, Dallas, TX 75252, USA; 2 Department of Veterinary Sciences, University of Turin, 10095 Grugliasco, TO, Italy; irene.viola@unito.it (I.V.); silvia.miretti@unito.it (S.M.); eugenio.martignani@unito.it (E.M.); 3 Department of Information Engineering, University of Pisa, 56126 Pisa, PI, Italy; stefano.giordano@unipi.it; 4 Department of Chemistry, Life Sciences and Environmental Sustainability, University of Parma, 43124 Parma, PR, Italy

**Keywords:** internet of things, propagation loss, smart agriculture, LPWAN, LoRa, remote sensing, link quality

## Abstract

The agricultural sector is amidst an industrial revolution driven by the integration of sensing, communication, and artificial intelligence (AI). Within this context, the internet of things (IoT) takes center stage, particularly in facilitating remote livestock monitoring. Challenges persist, particularly in effective field communication, adequate coverage, and long-range data transmission. This study focuses on employing LoRa communication for livestock monitoring in mountainous pastures in the north-western Alps in Italy. The empirical assessment tackles the complexity of predicting LoRa path loss attributed to diverse land-cover types, highlighting the subtle difficulty of gateway deployment to ensure reliable coverage in real-world scenarios. Moreover, the high expense of densely deploying end devices makes it difficult to fully analyze LoRa link behavior, hindering a complete understanding of networking coverage in mountainous environments. This study aims to elucidate the stability of LoRa link performance in spatial dimensions and ascertain the extent of reliable communication coverage achievable by gateways in mountainous environments. Additionally, an innovative deep learning approach was proposed to accurately estimate path loss across challenging terrains. Remote sensing contributes to land-cover recognition, while Bidirectional Long Short-Term Memory (Bi-LSTM) enhances the path loss model’s precision. Through rigorous implementation and comprehensive evaluation using collected experimental data, this deep learning approach significantly curtails estimation errors, outperforming established models. Our results demonstrate that our prediction model outperforms established models with a reduction in estimation error to less than 5 dB, marking a 2X improvement over state-of-the-art models. Overall, this study signifies a substantial advancement in IoT-driven livestock monitoring, presenting robust communication and precise path loss prediction in rugged landscapes.

## 1. Introduction

The pervasive integration of the internet of things (IoT) across various domains has facilitated the proliferation of interconnected devices, revolutionizing numerous industries, notably agriculture [1,2]. Amidst the sphere of smart agriculture, characterized by the dominance of IoT technology, a particularly notable segment pertains to livestock farming [3,4]. In this domain, managing animal positioning for their welfare becomes challenging due to inadequate supervision, necessitating the monitoring of livestock positioning and behavior in rugged terrains through IoT devices.

In regions with rugged terrain, farmers encounter a significant obstacle in monitoring sheep. The challenging landscape, characterized by steep slopes, limits access to alpine pastures, making it difficult to oversee the livestock. Furthermore, these mountainous areas are often expensive and impractical to fence due to the terrain. Consequently, there is an increased risk of sheep injuring themselves, particularly when coupled with infrequent monitoring, leading to potentially life-threatening accidents. To tackle this issue, herders require tracking systems capable of remotely locating their sheep. Reliability over extended periods is crucial for such systems. Typically, the main concern for maintenance revolves around battery replacement or recharging. However, performing maintenance during deployment is unfeasible for herders as it interferes with the primary purpose of the tracker. Therefore, energy efficiency becomes paramount in the design of such tracking devices, necessitating optimizations at both software and hardware levels.

Selecting an appropriate communication technology, coupled with optimal update rates, helps minimize power consumption during data transmission by the sensor nodes. Additionally, durability is imperative for animal trackers to withstand harsh environmental conditions during deployment. While many studies are conducted in controlled environments, they may not accurately reflect real-world application scenarios. Thus, on-site evaluations are indispensable for assessing system performance accurately.

In the realm of livestock farming applications, pivotal technological requirements encompass low-power consumption, cost-effectiveness, extended coverage, and positioning accuracy. Addressing these needs, Low-Power Wide Area Networks (LPWANs) have gained traction, boasting attributes like prolonged lifespan, cost-efficient chip integration, and expansive coverage. Prominent LPWAN technologies like Sigfox [5], Long-Range (LoRa) [6], and Narrowband-IoT (NB-IoT) [7] offer diverse capabilities, augmenting connectivity while maintaining energy efficiency. Amidst various technological options, LoRaWAN emerges as a widely embraced LPWAN technology, garnering significant attention across both academic research and industrial sectors. Our particular focus is directed towards LoRa and LoRaWAN technologies due to the prevalent absence of telecommunication coverage in rural and mountainous regions, compounded by challenges related to troubleshooting and maintenance. The cost-effective and energy-efficient nature of LoRa renders it highly suitable for connectivity purposes, especially in the realm of monitoring and control operations. LoRaWAN, distinguished by its low-power, long-range communication protocol, adeptly fulfills connectivity needs across expansive grazing territories while upholding robustness through low energy consumption. This contrasts with cellular-based alternatives such as 3G, 4G, and 5G, which offer wider coverage but necessitate high power consumption.

Optimizing the effective deployment of LoRaWAN entails radio planning activities, particularly crucial in challenging environments such as mountainous pastures. These activities are vital due to formidable radio propagation dynamics influenced by physical barriers shaping electromagnetic wave behavior. Although LoRaWAN enables long-distance connections, real-world implementations can exhibit significant variations in communication range. The power attenuation of the link, known as *path loss (pl)*, varies when an end device is deployed in different positions from a gateway due to several factors, encompassing terrain features and the diverse array of land-cover types such as trees, grasslands, and buildings along the transmission path. Developing a precise path loss model is paramount for LoRaWAN applications as it directly impacts the likelihood of successful packet transmission [8]. Thus, accurate prediction of path loss associated with a LoRa gateway prior to deployment holds the potential to enhance LoRaWAN coverage by strategically choosing gateway locations that minimize such loss.

This study proposes a deep learning framework tailored to precisely estimate path loss in long-range LoRa links. By harnessing publicly available remote sensing images, the framework adeptly identifies detailed land-cover distribution along the link, a critical factor significantly impacting path loss. Our study generates precise land-cover maps by leveraging the distinct spectral responses of various land-cover categories and employing supervised classification techniques. In our approach, we utilize pixel-based support vector machines (SVMs). The selection of land-cover classes is influenced by factors such as the target area’s presence, its relevance in characterizing LoRa links, and the potential for separation in multispectral images. The initial stage of our path loss estimation involves land-cover classification, offering detailed insights into the land-cover characteristics encountered by the LoRa link, as illustrated in Section 4.2.1.

Given the complexity of land-cover effects on path loss, the utilization of deep learning methodologies [9] becomes imperative for modeling the impact of a specific land-cover distribution on path loss accurately. Rather than treating a LoRa link environment as a whole, we propose viewing them as an ordered sequence of short, uniform links identifying detailed land-cover specifics for each LoRa link via remote sensing images. Following land-cover classification, we extract the land-cover sequence for the desired link region and utilize it as input for a deep neural network (DNN). Specifically, we employ Bidirectional Long-Short-Term-Memory (Bi-LSTM) units, enabling the network to analyze the sequence and build a path loss model from assessments in the area of interest. This deep learning model inherently captures the relationship between land-cover types, their sequence, and resultant path loss. Post-training, the model can be seamlessly applied to regions exhibiting similar land-cover compositions, requiring minimal data collection and model fine-tuning.

The deep learning framework utilized in this study originates from data acquired through experimental measurements conducted in mountainous environments. Our deployed LoRaWAN system comprised a single gateway and eight mobile LoRa end devices affixed to sheep traversing a 4 km × 4 km area within the mountainous region. These nodes periodically transmitted data packets containing location information as the sheep moved. Despite leveraging the mobility of these LoRa end devices, efficient recording of thousands of LoRa links across diverse locations poses challenges due to the non-uniform distribution of collected data within the areas of interest. The dataset encompasses over 35,876 packets logged by a gateway sent by eight mobile LoRa end devices. We aimed to delve into the stability of LoRa link performance in spatial dimensions, evaluating the extent of reliable communication coverage achievable by gateways in mountainous environments. Additionally, the experiment explored path loss estimation, revealing a mean error of 4.97 dBm, which is twice as smaller than the state-of-the-art models.

Key contributions of this study encompass the following:

- Description of the hardware design utilized for experimental evaluation.
- Empirical analysis investigating the impact of land-cover sequences on path loss within a real LoRaWAN system. This includes introducing a deep learning approach employing adaptive Bi-LSTM to explore the correlation between path loss and various land-cover types and their ordered sequence.
- Measurement of spatial link dynamics and calculation of coverage areas using sparsely received LoRa packets.
- Implementation and performance assessment of a deep learning path loss prediction model within an actual LoRaWAN deployment, showcasing experimental results with a mean error twice as small as that of existing state-of-the-art models.

The subsequent sections of this paper are organized as follows. Section 2 delves into related works, followed by Section 3, which focuses on the background and motivation of our study. Section 4 introduces the system design of the path loss prediction model. The hardware used for the LoRaWAN system is also presented in this section. Section 5 presents the results and discussion. Lastly, Section 6 concludes the paper with some final remarks.

## 2. Related Works

Within this section, we provide a concise overview of pertinent research of LoRaWAN field studies. Subsequently, we delve into the exploration of related studies concerning land-cover and propagation models.

### 2.1. LoRaWAN Studies in the Field

In recent years, various studies [10,11,12,13,14,15] have delved into assessing the performance of LoRaWAN in real-world settings. The authors in [10] focused on understanding the scalability bounds of a typical LoRa cell. The researchers achieved this by creating a LoRa tracker module embedded in a bike, testing it extensively across a large area within Palermo city. Notably, they achieved a maximum coverage distance of 7.3 km. Yang et al. [11] conducted a comprehensive analysis of LPWAN link dynamics, examining both macro and micro aspects. They performed extensive measurements in a deployment spanning a 2.2 × 1.5 km^2^ area for one month. Their network consisted of 50 nodes with three gateways, and they proposed a method to tackle link degradation issues. Another study in [12] provided subtle details regarding the design, development, and evaluation of a wildlife monitoring application using LoRa for IoT animal repelling devices. Their assessment involved testing LoRa transmission technology in forested areas operating within the 433 MHz and 868 MHz bands, highlighting its performance. Chall et al. [13] investigated the LoRaWAN radio channel specifically in the 868 MHz band. They conducted broad measurement studies in both indoor and outdoor environments, spanning urban and rural locations in Lebanon. Xu et al. [14] studied the behavior of LoRa links and energy profiles through the deployment of ten stationary and two mobile LoRa end nodes. These empirical studies revealed diverse conclusions about LoRa coverage, indicating that path loss tends to increase with communication distance at varying rates across different environments. Consequently, modeling the environmental impact on path loss has become a central focus in the design of path loss models.

### 2.2. Land-Cover and Propagation Models

Extensive field measurements have taken place in diverse indoor and outdoor environments, exploring cellular and wireless sensor networks’ path loss characteristics. Path loss is influenced by multiple variables including distance, frequency bands, average antenna elevations, and geographical features such as terrain, obstacles, buildings, hills, mountains, and human presence. The International Telecommunications Union (ITU) [16], Okumura–Hata [17], Cost-Hata [18], and other research institutions and standard bodies have produced a number of path loss models tailored for outdoor settings within the [800–1800] MHz and [2.5–5] GHz frequency bands. Empirical models like Okumura–Hata offer ready-to-use formulas adaptable to different settings. However, these models may yield inaccurate predictions when applied directly to new environments with distinct path loss patterns. A distinctive approach by Bor et al. [19] employs on-site measurements to calculate absolute path loss via path loss exponent, deviating from the free space path loss concept.

While widely used, these path loss models are unsuited for LoRaWAN networks operating in the 868 MHz band. Limited studies have evaluated radio propagation models’ performance in diverse regions like Lebanon [13] and Finland [20], but these models often rely on regional environment information for predictions. Such approaches, assuming uniformity in deployment areas, fail to account for anisotropic land-cover compositions along LoRa links. The authors in [21] and SateLoc [22] utilized remote sensing to quantitatively analyze land-cover compositions along LoRa links. However, Demetri et al. [21] chose an Okumura–Hata formula based on dominant land-cover, without directly incorporating land-cover effects into predictions. SateLoc [22] divided links into segments characterized by distinct land-covers, applying the Bor model along with associated path loss exponents. However, reordering these segments within a link yields consistent results, limiting the model’s adaptability. These existing models fall short in harnessing fine-grained environmental data and struggle to transition effectively to new contexts due to their fixed environmental modeling.

In contrast, our approach adopts a model based on a Recurrent Neural Network (RNN) to capture the intricate interplay between path loss, land-cover types, and their sequence along the path. This choice promises enhanced path loss estimation accuracy, while the use of raw environment data and extremely general RNN models enhances model transferability, overcoming the limitations of the conventional physical path loss models.

Additionally, we conducted an analysis of the spatial attributes of LoRa links and offered a more comprehensive study of coverage areas compared to previous research endeavors.

## 3. Background and Motivation

To calculate path loss, the received signal strength $\left(P_{RX}\right)$ is estimated, considering both the signal-to-noise ratio (SNR) and the received signal strength indicator (RSSI). When SNR > 0, $P_{RX}$ equals RSSI; otherwise, $P_{RX}$ is derived by adding SNR to RSSI. The path loss calculation is as follows:

(1) $$PL=P_{TX}-P_{RX}+G_{TX}+G_{RX}-L_{TX}-L_{RX}$$

Here, $P_{TX}$ represents the transmit power in dBm, while $G_{TX}$ and $G_{RX}$ denote the antenna gains of the transmitter and receiver, respectively. $L_{TX}$ and $L_{RX}$ signify negligible losses attributable to cables, as postulated within this context. Shadowing is described by a zero-mean Gaussian variable characterized by a standard deviation, σ, quantifying the divergence between observed and anticipated path losses.

Upon receiving a LoRa packet, the gateway provides essential metrics such as RSSI and SNR. While RSSI is a prevalent signal attenuation indicator in wireless sensor networks [23,24], its accuracy in LoRaWAN can be compromised due to the superimposition of LoRa signals and various noise sources, particularly below the noise floor. To address this, we employ Expected Signal Power (ESP), outlined in [21] as a more reliable metric. ESP delineates the actual received signal power over long-distance transmissions, calculated by the following equation:

(2) $$ESP=RSSI+SNR-10{log}_{10}(1+10^{0.1SNR})$$

Here, SNR is measured in dB, while the other terms are expressed in dBm. One prevalent model used to estimate signal path loss is Free-Space Path Loss (FSPL).

This model describes path loss in an ideal scenario without obstacles or multipath effects, as given by the following formula:

(3) $$FSPL=20\log\left(d\right)+20\log\left(f\right)-27.55$$

Here, *d* represents distance in meters and *f* stands for frequency in MHz.

The fundamental free space model establishes a foundational reference point for path loss measurement, considering unobstructed line-of-sight (LOS) conditions between transmitting and receiving points. Meanwhile, the logarithmic distance path loss model, extensively used in outdoor scenarios, proposes an exponential path loss variation linked to the distance between points. In contrast, the ITM Longley–Rice path loss model adopts a comprehensive approach, accounting for several factors such as terrain, obstacles, frequency, and weather conditions. An empirical approach, the Okumura–Hata model, draws insights from extensive measurements across the 200 MHz to 2 GHz frequency range, primarily suited for rural regions. It assumes minimal dominant obstacles between the base station and mobile unit, as well as gradual changes in terrain profile. 

It is essential to acknowledge that these models were originally formulated within the context of cellular and wireless sensor networks, incorporating specific limitations concerning antenna heights and terrain configurations. In practical scenarios, achieving true free space conditions is challenging, and the free space path loss serves as a basic estimate for path loss in real-world LoRa deployments. However, it is important to note that the actual path loss of LoRa connections in real-world settings is greatly affected by environmental attenuation. Models that take environmental factors into account require access to environmental data. For example, the traditional physical models such as the Okumura–Hata and Bor models typically rely on two methods: (1) empirical estimations drawn from experience or (2) on-site measurements. These approaches are often resource-intensive, particularly for long-distance situations, and primarily offer generalized environmental insights to certain regions. Even if two LoRa connections exist within the same deployment area but traverse distinct types of terrain, they might still be treated as if they experience the same environment by utilizing identical formulas or path loss exponents. Specific terrain types are outlined in Table 1, and these distinct terrains lead to varying path loss effects, underscoring the heterogeneous nature of LoRa connections. Consequently, accurately estimating LoRa path loss necessitates comprehensive per-link environmental information. To address this, we conducted an empirical study leveraging measurements from our LoRaWAN system deployed in mountainous pastures in northern Italy. The study aims to establish patterns governing how different land covers influence path loss. 

Remote sensing techniques can be used to identify different types of land cover from multispectral images of large geographical areas. This can be done by extracting distinctive features from the images and using machine learning models, such as SVM and random forests (RFs). The integration of remote sensing into physical models can help to enhance the accuracy of path loss estimation by shifting from a regional environmental approximation to individual link-based models. This is because remote sensing can be used to obtain detailed information about the environment along the path of the signal, such as the types of land cover and the presence of obstacles. 

However, some studies have neglected the impact of specific land-cover types or the sequence in which they appear along the path. For example, the authors in [21] used the Okumura–Hata model to estimate path loss, but they only considered the dominant land-cover type. This approach can lead to inaccurate results, as the path loss can be significantly affected by the presence of obstacles, even if they are not the dominant land-cover type, with the Okumura–Hata model acting as a bottleneck. Similarly, SateLoc [22] divided the path into segments and aggregated the path loss for each segment independently. This approach does not take into account the order dependence of the segments, which can also lead to inaccurate results. Even when the segment order is altered, the outcome remains unchanged. It has been shown in the literature that the order of land covers along a connection can have a significant impact on path loss, even if the type of land covers themselves do not change. This is because obstacles closer to the end device are more likely to obstruct the signal. For example, a tree that is located directly between the end device and the gateway will have a much greater impact on the path loss than a tree that is located further away.

The path loss of a LoRa connection can be conceptualized as a result of traversing a sequence of short links; the order of the short links sequence implicitly influences the path loss. This inherent influence cannot be captured by conventional physical path loss models. To address this challenge, we resorted to RNN, a prevalent architecture within deep neural networks (DNNs), which are well-suited for this task as they can handle sequence data and capture the order dependence.

## 4. System Overview

In this section, we describe the hardware for the LoRaWAN measurements. We also detail the system design of the path loss prediction model.

### 4.1. Considered Hardware

**Figure 1:**
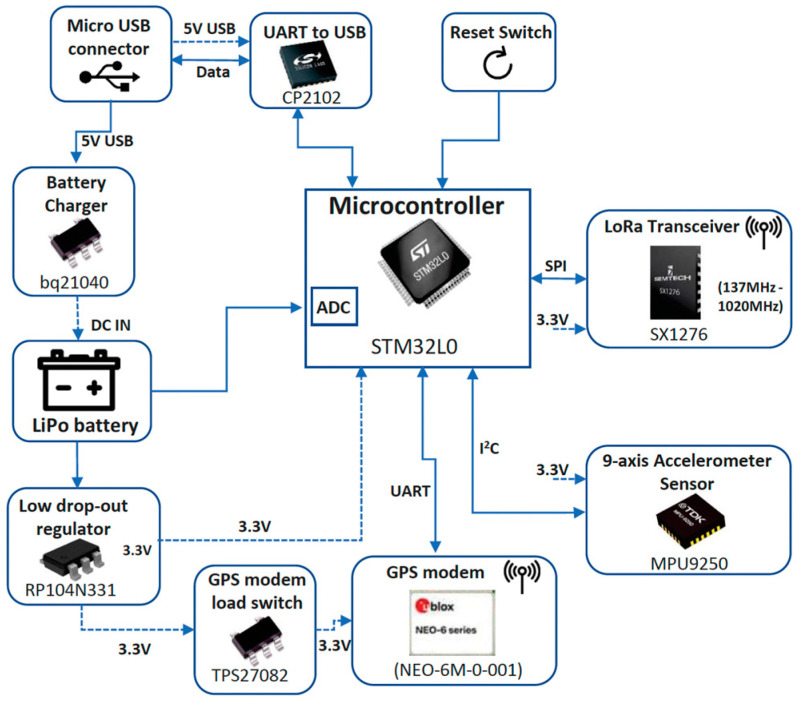
IoT device block scheme.

The end device (ED) was built around an STM32L designed by STMicroelectronics [25], featuring a 32-bit ARM Cortex M3-based architecture optimized for applications requiring low-power consumption. The ED comprised of several key components including a microcontroller, a global positioning system (GPS) modem, a UART to USB interface, and a LiPo battery among others, as demonstrated in Figure 1. Specifically, the microcontroller’s capacity for low-power run mode, specified with *μA* consumption, renders it an ideal choice for battery-powered scenarios. Additionally, it features multiple UART channels, two distinct serial peripheral interface (SPI) ports, and two inter-integrated circuit (I^2^C) interfaces. 

For debugging and programming purposes, the microcontroller supports a USB 2.0-compliant interface via a CP2102 UART to USB circuit provided by Silicon Labs, Austin, TX, USA [26]. This interface functions as a communication port “COM” for computer applications and draws power from the USB bus at 5 V, which is available through the micro-USB connector. Power was supplied by a 3700 mAh lithium polymer (LiPo) battery, with its voltage readings attainable through the microcontroller’s analog-to-digital converter (ADC). 

The ED additionally integrated an SX1276 module [27] from Semtech, designed as a long-range transceiver leveraging Semtech’s proprietary spread spectrum communication technology, facilitated through LoRaWAN. The connection between the LoRa module and the microcontroller was established via *SPI*, operating at a 3.3 V. Remarkably, the SX1276 exhibits exceptional sensitivity level exceeding −148 dBm, facilitated by an inexpensive crystal, thereby making it cost-effective and readily accessible in the market. The ED was also equipped with a nine-axis accelerometer, encompassing a three-axis gyroscope, three-axis accelerometer, and three-axis magnetometer. These components collectively offer comprehensive data on accelerations across all three axes as well as rotations around each axis. Additionally, the embedded GPS component from Ublox, operating at 3.3 V was integrated into the ED architecture for the purpose of ascertaining the ED’s geographical coordinates. This GPS was powered through a TI TPS27082 [28] load switch from Texas Instruments, which managed the power supply. Communication between the GPS and the microcontroller occurred via UART.

For communication purposes, we employed a Laird outdoor gateway due to its low cost and reliability. It is an eight-channel gateway built on the Semtech industry standard and can offer a secure, scalable LoRaWAN solution for complete management over a private LoRaWAN network. The LoRaWAN gateway was interconnected with an ADSL router, facilitating internet connectivity, and subsequently connecting to the Amazon web services (AWSs). This gateway, capable of receiving LoRa frames across a range of signal strengths, was linked to the AWS-provided network server “AWS IoT Core for LoRaWAN”. Both the gateway and the EDs utilized an omni-directional dipole antenna, boasting a 2 dBi gain. Upon receiving each data frame, the gateway yielded crucial parameters such as RSSI, SNR, and the payload message. These parameters were logged on to the AWS server for subsequent analysis and processing, utilizing InfluxDB to store the data. 

Given the considerable variation in LoRa performance depending on selected transmission parameters, as established in [29], the following parameter configuration was chosen:

- Bandwidth (BW): The transmission frequency range is defined by the BW parameter, which ranges from 7.8 kHz to 500 kHz. Widening the bandwidth decreases receiver sensitivity but enhances data rate due to reduced Time-on-Air (ToA). Our experiment employed a BW setting of 125 kHz.
- Transmitted Power (PTX): For LoRa end devices operating in the 433 MHz and 868 309 MHz bands, the maximum effective isotropic radiated power (EIRP) in the default setting is 12.15 dBm and 16 dBm, respectively. Our experiment adhered to the highest permissible PTX within the EU 868 MHz band, aligned with the LoRa device’s approved duty cycle of 1%. This led to a selection of P_TX_ = 14 dBm.
- Carrier Frequency (CF): A number of factors led to the adoption of the 868 MHz frequency. While path loss is lower in the 433 MHz band compared to 868 MHz, the 433 MHz band enforces a maximum transmitting power of 10 dBm. Furthermore, at 433 MHz, antenna dimensions are larger for a given radiation efficiency. Lastly, due to its narrower bandwidth, the 433 MHz band accommodates fewer communication channels [30].
- Spreading Factor (SF): This factor indicates the number of bits sent in each LoRa symbol. SF varies from 7 to 12, resulting in distinct ToA and receiver sensitivity values. Higher SF, such as SF = 12, corresponds to reduced receiver sensitivity [31], enhancing the link budget. The transmission rate is halved when SF is increased by one unit, which doubles the channel usage, energy consumption, and transmission time (sleep time). The relationship between LoRa transmission ToA and the employed LoRa parameters is expressed as ToA = 2SF/BW.
- Coding Rate (CR): CR equals 4/(4 + *n*), with *n* ∈ {1, 2, 3, 4}. To minimize ToA, CR = 4/5 was selected.

To meet the requirements of deep learning time series acquisition, the data acquisition device needs to satisfy the following demands:

- The device should operate reliably over extended periods, consistently collecting data at uniform time intervals. This minimizes data loss and ensures the highest accuracy and reduced errors within the deep learning model.
- Its hardware design and communication method should be flexible. To be adapted to more applications for time series data acquisition it should have the ability to access and expand the sensors with common interface.

### 4.2. Path Loss Prediction Model

**Figure 2:**
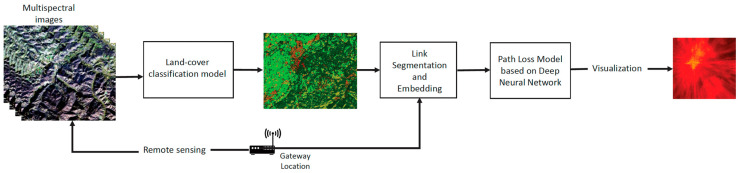
System design overview.

In this subsection, we outline the proposed design of our deep learning-based system, devised to yield precise path loss estimates by leveraging land-cover classification and their sequence along propagation paths. Our methodology entails fusing land-cover recognition with path loss modeling. A comprehensive depiction of our path loss prediction model, hinging on deep learning, is presented in Figure 2. Our system architecture was compartmentalized into three distinct blocks: land-cover map classification, link segment and embedding, and the DNN-based path loss model. Each pixel in the land-cover map is the class label that represents the true land-cover type in the real map. Then, link segmentation and embedding produces a formalized sequence by segmenting any LoRa link from an end node to the gateway into multiple short links of the same length and embedding each short link into one element in the sequence based on the land-cover map. Moreover, our path loss model based on DNN takes the sequences together with experimental specific parameters as input and predicts corresponding path loss such that the ESP received by the gateway can be calculated.

#### 4.2.1. Extracting Land-Cover Maps from Multispectral Images

Our methodology involved the automatic extraction of land-cover classes from multispectral images acquired from the US Geological Survey (USGS) Landsat series of Earth Observation satellites, which are accessible through the Google Earth Engine (GEE) platform. To derive precise land-cover maps, we leveraged the distinctive spectral responses exhibited by various land-cover classes, employing supervised classification techniques rooted in machine learning. Specifically, we focused on kernel-based methodologies, particularly pixel-based SVM [32,33] chosen for their advantageous attributes, including robust generalization capabilities, excellent classification accuracy, and a comparatively streamlined design with minimal control parameters. 

We delineated the land-cover classes as seen in Table 1 through the following criteria, considering their presence in the target area, their relevance in characterizing LoRa links, and the potential to discriminate them in the multispectral images. Our initial step involved generating a land-cover map classification, a crucial process for comprehensively interpreting the land-cover information embedded within the LoRa link. Employing an automated system, we assigned each 10 m×10 m pixel in the images to the most suitable land-cover class according to predefined standards relying on spectral features, including raw pixel spectral values, the Normalized Difference Vegetation Index (NDVI), and the Normalized Difference Water Index (NDWI) for individual pixels. 

We use non-linear approaches to solve this problem, which was complicated by several non-linearly separable classes. In particular, we used the SVM’s Radial Basis Function (RBF) kernel [34], which takes the feature vector to predict whether an area pertains to a specific land-cover type. Similar to the approach described by Demetri et al. [21], the workflow necessitated a training set of manually labeled reference pixels linked to land-cover classes via image interpretation. During the classifier’s learning phase, this dataset played a dual role in the model selection and SVM training. The SVM was used to automatically create the land-cover map after it had been trained across all images that were taken into consideration.

To ensure accuracy, each image underwent independent classification. This involved collecting image-specific datasets containing 200 samples for training and 100 samples for testing for each land-cover class. The test samples were used to assess the classification accuracy for each image. By using a grid-search model selection based on five-fold cross-validation, the critical model selection phase found application-specific optimal SVM tuning parameters, such as the regularization parameter and the RBF kernel width. The goal was to accurately discriminate classes and minimize expected generalization errors within predetermined ranges for the values of the regularization parameter and the RBF kernel width. Using conventional tools and methodologies, the optimal regularization parameter and RBF kernel width values were found through cross-validated classification accuracy and used in future SVM training and classification processes.

#### 4.2.2. Link Segment and Embedding

**Figure 3:**
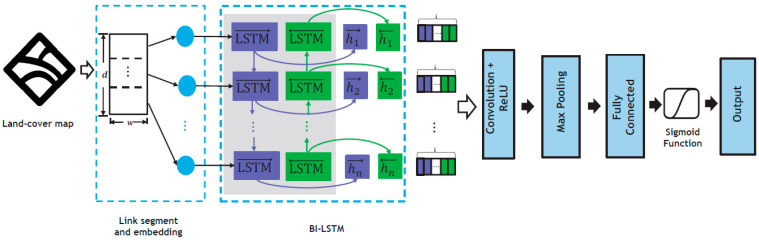
Path loss prediction model using deep learning with Bi-LSTM architecture.

**Figure 4:**
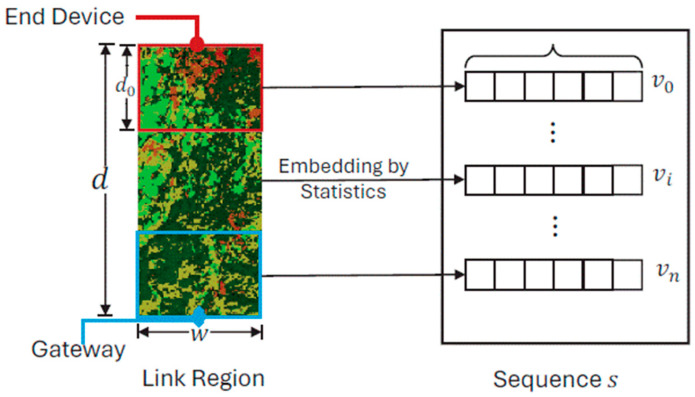
Link segment and embedding.

The subsequent phase, link segment and embedding, revolved around capitalizing on the comprehensive environmental insights garnered from the land-cover classification. We formalized a deep learning path loss model that was built upon this detailed environmental information. Instead of considering a mere “line”, we opted for a more encompassing approach. Specifically, we chose a rectangular region from the land-cover classification map that linked the gateway and the end device. In this study, making a line was hard to determine, because the path from the gateway to the end device was usually a non-LOS path. The choice of choosing a rectangle mitigated the impact of potential misclassifications on the accuracy of the sequence. In addition, the rectangular area indicating the land-cover map shown in Figure 3 offered fault tolerance by capturing a broader land-cover representation. The width of this rectangular was carefully chosen based on empirical data and experimental findings. The link segment of length d and width w was divided into smaller short links of length $d_{0}$ from the end device to the gateway, as shown in Figure 4. The granularity and length of the resulting sequence, determined by $d_{0}$, directly influenced the estimation accuracy. For each short-link region, we counted the proportion of pixels belonging to each of the land-cover types.

For instance, in a short-link region, ${sk}_{i}$, 0≤i<n, which contains $c_{k}$, 0≤k≤5 pixels for each land-cover type $l_{k}$. Then, each short-link region is embedded into a 1×6 vector $v_{i}$ by counting the proportion of six land-cover types as follows:

(4) $$v_{i}=\left[v_{i}^{0},v_{i}^{1},\;v_{i}^{2},v_{i}^{3},\;v_{i}^{4},v_{i}^{5}\right]$$

(5) $$\;v_{i}^{k}=c_{k}/\;\sum_{j=0}^{5}c_{j}$$

By concatenating these vectors for all short links, we obtain an ordered sequence $s=\left[v_{0},\;v_{1},\;\cdots ,\;v_{n-1}\right]$ representing the land-cover composition along the link. This sequence subsequently serves as input for the DNN, specifically a Bi-LSTM unit, for further analysis.

Thus, we obtained a structured representation by splitting LoRa links into several equidistant short links and embedding each short link into the sequence according to the land-cover map.

#### 4.2.3. DNN-Based Path Loss Model

**Figure 5:**
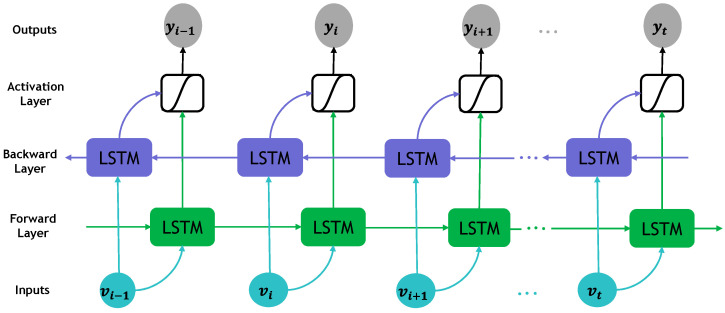
The information flow of Bi-LSTM. $v_{i-1}$, $v_{i},\;v_{i+1},\;v_{t},\;$are vectors in the input sequence, and $y_{i-1}$, $y,\;y_{i+1},\;y_{t},\;$are the output hidden states from different frames of Bi-LSTM.

In accordance with the schematic overview presented in Figure 2 of the system design, the sequence comprised of feature vectors was fed into the Bi-LSTM unit to disentangle order dependencies. The architectural layout of the path loss model based on DNNs is depicted in Figure 3. Capitalizing on the temporal dimension, which aligns with the distance parameter in our case, allowed for the flow of information from the sequence’s start to its end. This characteristic was particularly advantageous in estimating the path loss at the gateway, situated at the sequence’s last frame, accounting for attenuation throughout the sequence. To tackle the limitation of RNNs in learning long-term dependencies, we embraced the Bi-LSTM units [35,36]. Figure 5 depicts the construction of a Bi-LSTM.

The Bi-LSTM architecture incorporates bidirectional information flow, illustrated by both purple and green arrows in Figure 5. This network comprises a forward layer and a backward LSTM layer, as pioneered by Schuster and Paliwal [36], allowing it to extract temporal patterns from both past and future data. By connecting both forward- and backward-pass LSTM networks to the same output layer, this design enables the capture of comprehensive temporal information. This bidirectional approach ensured that land-cover information from both the sequence’s commencement and conclusion was effectively captured.

Additionally, an activation layer is commonly integrated to enhance the learning capability of the training model. This layer utilizes specific activation functions, such as mathematical formulas governing neural network output computation. These functions can resemble step functions, dictating when a neuron output activates or deactivates based on predefined thresholds. Activation functions fall into three categories: binary step, linear, and non-linear. In our study, we employed a non-linear activation function to augment model performance.

The output from the Bi-LSTM unit was then channeled through convolution layers, enabling the extraction of local features and context dependencies. Introducing non- linearity via Rectified Linear Unit (ReLU) layers enhanced the model’s expressive power. Subsequent max pooling down-sampled output features, effectively reducing dimensionality. These features were then linearly mapped to path loss within the fully connected layer. This extensibility equipped our network to accommodate various LoRa link attributes, such as weather conditions, temperature, and more, facilitating quantitative analysis of additional influencing factors. It is vital to consider that path loss possesses constraints; it cannot fall below zero and must adhere to the maximum link budget imposed by the highest transmitting power and end device sensitivity. Therefore, we applied a sigmoid function to curve our final estimation, ensuring that path loss values were confined within a range of 0 to 1, enhancing training convenience. This approach facilitated scaling of the estimation with the upper boundary limit to yield expected path loss, with values exceeding the boundary indicating packet delivery failure. To evaluate the model, we trained and updated the model using a back-propagation algorithm as an optimizer.

Given the upper boundary of 160 dBm, our system can be tailored to the specific constraints of different countries/regions. Our system design facilitates effective adaptation to new environments by employing the following strategies:

- We refrained from manual feature selection and instead utilized a sequence restructured from genuine land-cover maps along with additional variables as inputs. As a result, our model is able to obtain a mapping that is quite similar to the principles of signal propagation.
- During the training process, we made a deliberate effort to incorporate training data encompassing diverse link distances and variations in land-cover compositions. This approach ensures that our training dataset effectively covers a wide spectrum of the feature space.
- Our path loss model adopts a Bi-LSTM-based DNN architecture. Neural networks trained on comprehensive historical datasets can be fine-tuned using a smaller dataset containing new data, enabling the model’s weights to be adjusted to new observations. As a result, fine-tuning the model with a limited amount of data from a new environment can yield superior accuracy compared to the original model. This stands as an advantage over a lot of other machine learning-based models that necessitate retraining from scratch using fixed data and do not guarantee improved outcomes.

## 5. Results and Discussion

### 5.1. Experimental Environment and Collected Dataset Overview

#### 5.1.1. Experimental Environment

Our study took place within the mountainous pastures in the north-western Alps (Ormea, CN, Italy), situated at an elevation of 1340 m above sea level. This locale serves as a crucial source of seasonal forage for livestock. Characterized by modest hills with an elevation difference ranging from 80 to 100 m, the area is encompassed by forest, grassland, and farmland, and diminutive hills with a lack of buildings and other barriers.

In this dynamic landscape, the EDs traverse varying locations within the designated area. In other words, throughout the measurements, the gateway’s position remained stationary, while the EDs were relocated following the sheep movement within the mountainous pasture. The dataset gathered from this experimental deployment and evaluation in the northern pastures is crucial for training the path loss deep learning model and assessing its performance.

#### 5.1.2. Collected Dataset

We present here an overview of our collected dataset, covering a period from 15 July to 17 October. The transmission of packets was obtained in a periodic manner, and the payload from the LoRa EDs included crucial information such as GPS coordinates, timestamps, and sequence numbers. In addition, the gateway recorded the associated SNR and RSSI values. These logged data records, which made up a final dataset of over 35,876 records, could be extracted from the network server after packet reception. Moreover, we can compute essential metrics like the link distance d and the height difference h between the end device and gateway pair by decoding the GPS data embedded within the payloads. This process enabled us to derive additional contextual information regarding the spatial relationship and positioning between the EDs and their respective gateway.

### 5.2. Link Behavior Study

Two key metrics, namely the Packet Delivery Ratio (PDR) and ESP, serve as indicators for signal path loss across a physical channel to ensure reliable coverage within an area. Through a detailed examination of their distribution, we have devised a predictive model for PDR. This model correlates the computed ESP value of a position with the estimated PDR, enabling the determination of our LoRa system’s coverage for end devices at each position. Considering the mobility of the end devices, particularly in scenarios such as the movement of sheep, data packets are dispersed along diverse trajectories. Our main approach was to compute the PDR of a specific position by using all trajectories that pass the position according to their coordinates. This methodology allowed us to comprehensively calculate the PDR concerning the various paths traversed by the data packets due to the movement of the end devices.

#### 5.2.1. Overall PDR and ESP Distribution

**Figure 6:**
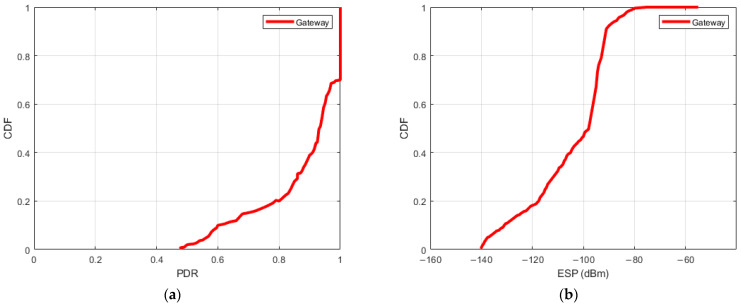
CDF of PDR and ESP observed at the LoRa Gateway. (**a**) Top–CDF of PDR, (**b**) Bottom–CDF of ESP.

We present the estimated PDR and ESP for various positions in relation to the gateway. Figure 6 portrays the Cumulative Distribution Function (CDF) of PDR, indicating that 60% of the links exhibit high reliability with a PDR exceeding 90% for the gateway. The remaining 40% of LoRa links exhibit variable behaviors, denoting intermediate link performance. Figure 6b displays the CDF of ESP derived from all recorded data packets. Notably, the minimum ESP registers at −140 dBm across all packets, aligning consistently with the reported sensitivity of SX1276 at −148 dBm [27]. Furthermore, approximately 80% of the gateway’s ESP values are from −140 dBm to −120 dBm, while the maximum ESP reaches −55 dBm. This observation underscores the deployment environment’s characteristics, indicating an unobstructed antenna path for the gateway. 

Figure 6 illustrates a notable disparity in distribution between PDR and ESP. This discrepancy highlights the distinct behavior wherein the robust noise tolerance characteristic of LoRa technology allows for a convergence in PDR distribution despite varying ESP levels. For instance, even with a low ESP, such as a median value of −125 dBm, there is an observable similarity in PDR distribution comparable to instances with higher ESP, like the median value of −89 dBm.

#### 5.2.2. Spatial PDR Distribution

**Figure 7:**
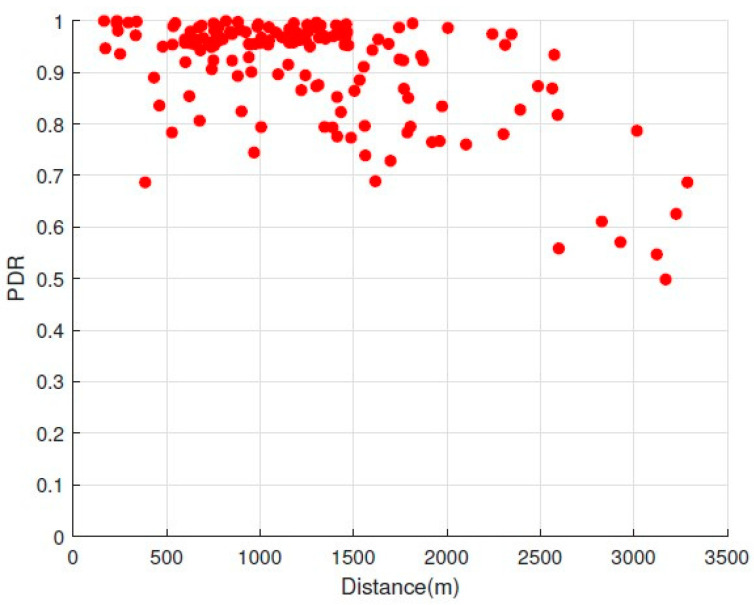
The spatial distribution for PDR and distance.

Our study involved analyzing the spatial distribution of PDR as it relates to the link distance. We divided the area into “positions” (i.e., 100 m×100 m block). For each position, we calculated the distance between its center and a gateway location. Then, we leveraged the GPS coordinates of each transmitted packet to compute the distance it traveled to reach the gateway. Figure 7 illustrates the spatial distribution of PDR. Upon analysis of Figure 7, we note that lower PDR values are dispersed across intermediate links at various distance levels.

#### 5.2.3. ESP-Based PDR Prediction

**Figure 8:**
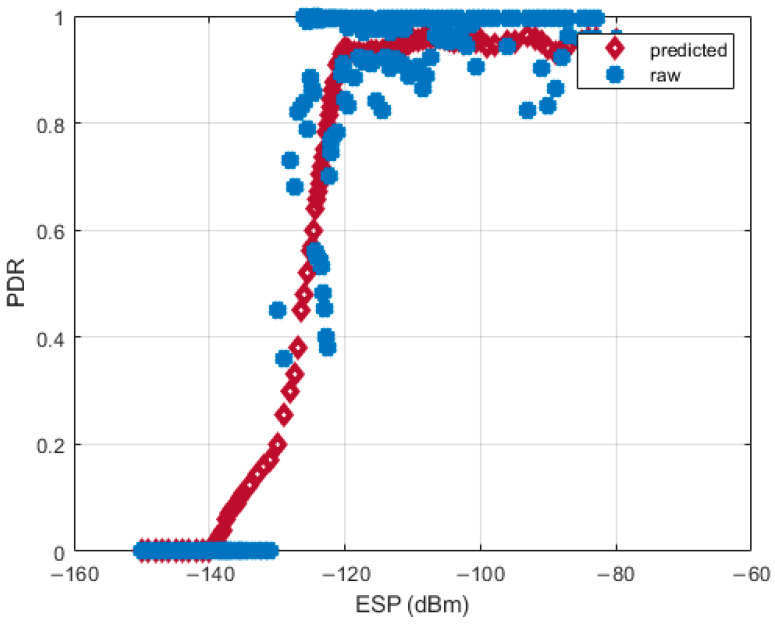
PDR against ESP at our gateway.

We developed a PDR prediction model with ESP as the input variable, based on the data we had previously generated for the PDR and ESP distributions. First, we calculated the average ESP for every data record that corresponds to a specific sheep position in the mountainous pasture region. This facilitated the creation of diverse PDR–ESP pairs based on measured PDR values for the covered areas. Next, we employed Gaussian process regression (GPR) [37] to predict PDR for uncovered areas solely based on their ESP values. To achieve optimal regression accuracy, we opted for an exponential kernel function and conducted a rigorous fitting process, depicted in Figure 8. The statistical evaluation of our model achieved impressive performance, boasting a coefficient of determination (R2) of 0.78 and a root mean-square error (RMSE) of 0.129. Analyzing raw data pairs (represented by blue dots), we observed that for gateway ESP values below −131 dBm, the measured PDR plummeted to near 0. Conversely, when ESP exceeded −120 dBm, PDR reached high levels but rarely attained 100%. This can be attributed to the large temporal variance inherent in PDR and ESP measurements. Importantly, the predicted data points (illustrated by red diamonds) for uncovered areas aligned well with ground truth values. However, the model’s inability to accurately capture the dynamic nature of PDR in our LoRaWAN mobility system highlights limitations. Nevertheless, our findings demonstrate the potential of ESP as a reliable indicator for PDR prediction in such challenging environments.

### 5.3. Land-Cover Classification

**Figure 9:**
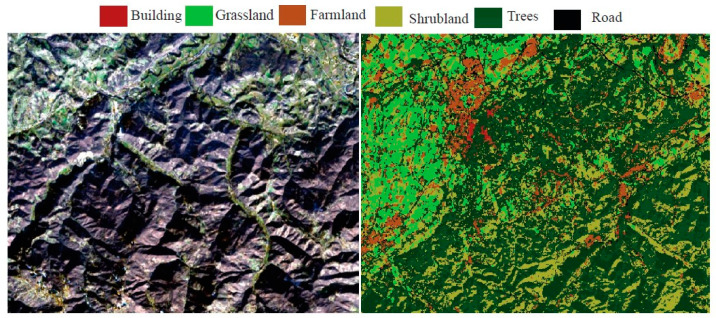
Map depicting the land-cover classification of a 4 × 4 km^2^ area of interest.

The land-cover classification analysis yielded an exceptional overall accuracy rate of 98% across diverse land-cover types. This high accuracy lends credibility to the resulting land-cover map as a reliable representation of the actual environmental conditions. Figure 9 provides a visual representation of the categorized land-cover. Distinct color coding differentiates various cover types. Predominantly, the area comprises trees, grassland, farmland, and shrubland encompassing a significant portion. Conversely, buildings and roads feature sporadically in select areas within the region. Water was not taken into account, as indicated in Table 1, because it is not present within the area of interest.

### 5.4. Path Loss Estimation

With the use of the PyTorch framework [38], our approach for the path loss model utilized a DNN. This model was trained on data collected during our LoRa experiment. To ensure the model’s effectiveness, we enforced the condition that identical inputs must yield identical outputs during the training phase, mitigating potential confusion within the model. This required careful data cleaning before training commenced. Since sheep movement generates continuous data, device locations obtained by the u-blox chip were plotted continuously on the map. 

Given the 10-m resolution of the multispectral images employed, each 10 m×10 m area on the ground is represented by a single pixel. This common practice in remote sensing and cartography facilitates managing large areas and maintaining consistent map detail. However, transforming GPS coordinates to map pixels can lead to multiple locations within the same pixel having different ground truth path loss values. In order to eliminate this duplication and create a distinct ground truth for every input, we computed the average path loss for measurements made within each pixel. To train and evaluate our path loss model, we split the dataset into a training set and testing set by 9:1. Since our model’s principle is sequence processing, and the length of a sequence has significant impact on path loss, we separated our data into bins based on their sequence lengths before we split the dataset. This methodology ensured diversity in sequence lengths within the training set, mirroring the distribution in the testing set for a more robust and generalizable model. Based on empirical performance in subsequent experiments, we selected *d* = 3 and *w* = 7 for link segmentation and embedding, representing 30 m and 70 m, respectively. The model was trained with a learning rate of 0.0001 and a batch size of 16, and its performance was evaluated every 5 epochs. The Adam optimizer was also used for network training. 

We analyzed our path loss prediction model’s performance across different environments and compared it to state-of-the-art methods. To gauge accuracy, we conducted an evaluation comparing our model with benchmarks including the free space model, Bor model, Demetri model, and SateLoc model, as detailed in the related work section. All models were evaluated on the same test set by computing the absolute difference between their path loss estimations and the ground truth values. Table 2 summarizes the results. 

Remarkably, our path loss prediction model demonstrates exceptional accuracy, achieving an error rate of 4.97 dB. This performance surpasses existing models by at least 50%. Additionally, with a standard deviation of 4.13 dB, our model demonstrates consistent and stable estimation performance. Notably, while the Bor model exhibits slightly superior performance compared to other models, this is attributed to its path loss model being derived from fitting equations using our training data. Conversely, SateLoc relies on provided path loss exponents, while the Demetri model employs Okumura–Hata formulations based on Tokyo data. The divergence among datasets from distinct environments contributed to increased estimation errors. Despite this, our prediction model consistently outperformed results reported in the original papers. 

**Figure 10:**
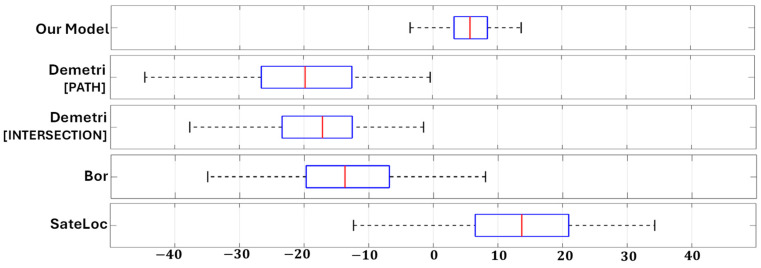
Distribution of the estimation errors on the test set.

Figure 10 presents a box plot visualizing the raw estimation errors of various models (excluding the free space model due to its disproportionately large error) across the full testing set. Our prediction model’s errors center around 0 dB, indicating no bias towards underestimating or overestimating of the path loss. In contrast, other models demonstrate significant offsets from 0. SateLoc exhibits the most significant deviation, further highlighting a considerable gap between the utilized path loss exponents and the actual rate of path loss increase with distance. Additionally, our model boasts a significantly narrower error distribution compared to others. The magnitude of its largest error remains below 10 dB, while 50% of errors fall below 5 dB. These findings underscore the superior accuracy and reduced variance of our path loss prediction model compared to existing methodologies.

### 5.5. LoRa Coverage Measurements

This section delves into the coverage analysis of our deployed gateway within the challenging mountainous environment. We define the coverage area as the region where the PDR exceeds 70%. To assess this, we split the area into 100 m×100 m grids (“positions”).

**Figure 11:**
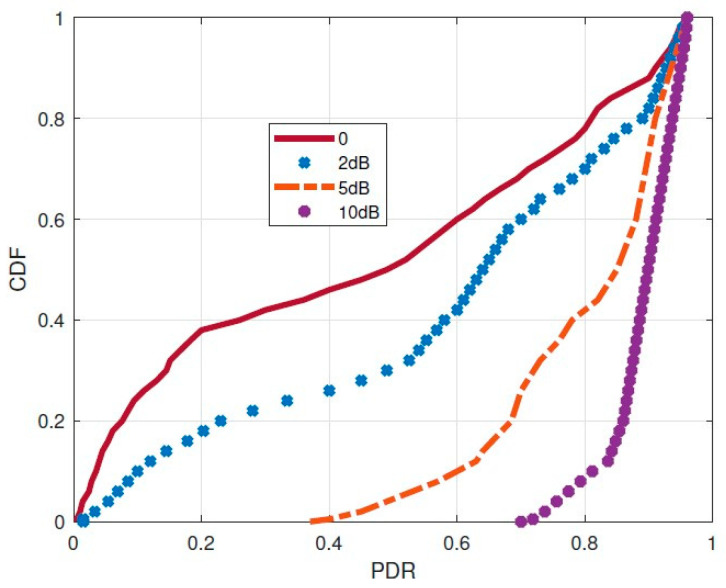
CDF of predicted PDR with different ESP gains.

For each position in the covered areas, we directly calculated the corresponding PDR based on our collected data. For the uncovered areas, we applied our path loss prediction model to estimate the average ESP for each position. Subsequently, we utilized the derived PDR–ESP regression model to predict the associated PDR based on the estimated ESP values. The correlation between SNR and ESP, demonstrated in Equation (2), holds significance in augmenting SNR gain for gateway coverage, a factor underscored in numerous studies. To quantify the ESP gains’ impact on coverage within our system, we manually introduced ESP gains per position and recomputed the corresponding PDR under this enhanced ESP. Randomly selecting ESP gains from 2 dB to 10 dB ensured fairness, generating the CDF of predicted PDR illustrated in Figure 11. As the additional ESP gains increase, a corresponding rise in PDR was observed, validating the efficacy of the SNR enhancement method.

Furthermore, Table 3 illustrates the utilization of enhanced PDR for calculating the coverage area, revealing consistent enhancement trends in our gateway’s coverage area with escalating ESP gains. For instance, with a 2 dB ESP gain, a notable 32.6% increase in coverage area is achievable. These outcomes suggest that due to the dynamic nature of link behaviors, a gateway’s coverage area tends to be irregular. Consequently, beyond deploying new gateways, optimizing gateway coverage by harnessing additional SNR gains from LoRa signals proves more effective in expanding coverage areas.

## 6. Conclusions

We conducted an extensive examination deploying a LoRaWAN system within mountainous terrains, employing a gateway and multiple nodes, specifically focusing on eight LoRa end devices attached to sheep. Over a three-month period, we diligently collected data packets within a 4 km×4 km mountainous area, revealing key insights into the dynamic behavior of LoRa link performance influenced by diverse land-cover types. Our findings unveiled the dynamic nature of LoRa link behavior in spatial dimensions, strongly influenced by diverse land-cover types. In addition, efficiently acquiring SNR gains from LoRa signals significantly expanded network coverage. Moreover, our study introduced a predictive path loss model tailored for LoRa links in mountainous pastures, deriving empirical insights into the relationship between link path loss and the specific land covers traversed. Leveraging freely accessible multispectral satellite images, we developed a remote sensing workflow facilitating quantitative analysis of land-cover compositions along the path of a LoRa link between the end device and gateway. Employing a recurrent neural network, specifically the “Bi-LSTM”, we captured the intricate interplay between path loss, land-cover types, and their sequence along the path. Comparative analysis against state-of-the-art models demonstrated the superior performance of our prediction path loss model, showcasing enhanced accuracy and granularity in path loss estimation while requiring minimal transferring training overheads. These results underscore the efficacy and advancement of our model in characterizing and predicting path loss in challenging terrains, offering notable advancements in LoRaWAN system performance analysis.

### 6.1. Challenges and Limitations

This study encountered several challenges that limit the broad applicability of the proposed approach. Scalability to diverse environments is hindered by variations in land-cover types and altitude. These variations can significantly impact the model’s ability to transfer effectively to new regions. Additionally, the inherent computational complexity of deep learning models comes into play. Training these models often necessitates vast amounts of data, leading to a trade-off between the length of the sequences being processed (for higher granularity) and the model’s overall efficiency. Furthermore, achieving high accuracy relies on carefully considering factors like land-cover types and their specific sequence along the path being analyzed. Future research efforts should address these challenges to broaden the applicability of this approach.

### 6.2. Future Directions

In future directions, we aim to extend our path loss prediction model to integrate seamlessly with other low-power wide area technologies, such as NB-IoT, to enhance coverage and reliability. Additionally, our focus will shift towards considering real-world deployment factors, including scalability and generalizability to diverse regions, to ensure practical implementation viability.

## Figures and Tables

**Table 1:** Types of land covers.

Trees	trees
Grassland	grazing lands
Farmland	crop fields
Water	rivers and lakes
Road	roads, paths
Building	buildings, huts
Shrubland	shrubland

**Table 2:** Estimation errors of absolute path loss across various models.

	Average (dB)	Standard Deviation (dB)
Our Model	4.97	4.13
Demetri [PATH]	19.23	8.71
Demetri [INTERSECTION]	18.71	8.85
Bor	12.53	9.69
Stateloc	15.94	10.83
Free-Space	42.92	8.76

**Table 3:** Coverage area under different ESP gains.

ESP Gains (dB)	0	2	5	10
Gateway Coverage Area (km^2^)	12.5	16.9	26.9	38

## Data Availability

The original contributions presented in the study are included in the article, further inquiries can be directed to the corresponding authors.
